# Herbivore Fronts Shape Saltmarsh Plant Traits and Performance

**DOI:** 10.1002/ece3.71360

**Published:** 2025-04-25

**Authors:** Serina S. Wittyngham, David S. Johnson

**Affiliations:** ^1^ Virginia Institute of Marine Science, William & Mary Gloucester Point Virginia USA

**Keywords:** consumer fronts, feeding fronts, plant defense, plant functional traits, plant‐herbivore interactions, *Sporobolus alterniflorus*, top‐down control

## Abstract

Herbivore fronts can alter plant traits (chemical and/or morphological features) and performance via grazing. Yet, herbivore‐driven trait alterations are rarely considered when assessing how these fronts shape ecosystems, despite the critical role that plant performance plays in ecosystem functioning. We evaluated herbivore fronts created by the purple marsh crab, *Sesarma reticulatum* , as it consumes the cordgrass, *Spartina alterniflora* , in Virginian salt marshes. *Sesarma* fronts form at the head of tidal creeks and move inland, creating a denuded mudflat between the tall‐form *Spartina* low marsh (trailing edge) and the short‐form *Spartina* high marsh (leading edge). We quantified *Sesarma* front migration rate, tested if *Sesarma* herbivory altered geomorphic processes and *Spartina* traits at the trailing and leading edges, and examined how these trait changes persisted through the final 8 weeks of the growing season. *Sesarma* front migration in our region is two times slower than fronts in the Southeast United States, and *Spartina* retreat rate at the leading edge is greater than the revegetation rate at the trailing edge. *Sesarma* fronts lowered elevation and decreased sediment shear strength at the trailing edge while having no impact on soil organic matter and bulk density at either edge. At the leading edge, *Sesarma* grazing reduced *Spartina* growth traits and defensive ability, and trait changes persisted through the remaining growing season. At the trailing edge, however, *Sesarma* grazing promoted belowground biomass production and had limited to no effect on growth or defensive traits. We show that herbivore fronts negatively impact saltmarsh plant traits at their leading edge, potentially contributing to front propagation. In contrast, plants at the trailing edge were more resistant to herbivore grazing and may enhance resilience through elevated belowground biomass production. Future work should consider herbivore‐driven plant trait alterations in the context of herbivore fronts to better predict ecosystem response and recovery.

## Graphical abstract

We show that *Sesarma reticulatum* fronts lower elevation and its direct grazing causes declines in *Spartina alterniflora* growth traits and defensive ability at the leading edge of the front. However, at the trailing edge, *Sesarma* razing increases *Spartina* belowground biomass, presenting an indirect pathway in which herbivory may enhance ecosystem stability.

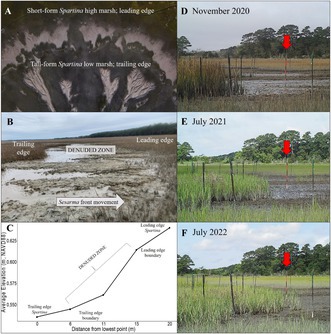

## 1 Introduction

Consumer fronts, dense aggregations of consumers bordering a resource, occur worldwide (insects in terrestrial grasslands: Lejeune et al. 2005; beetles in pine forests: Birt and Coulson 2015; urchins in kelp forests: Lauzon‐Guay and Scheibling 2007; green turtles in seagrasses: Gulick et al. 2021). As consumers deplete food and suitable habitat, fronts propagate through the landscape in search of additional resources (Silliman et al. 2013; Vu and Pennings 2021), shaping primary and secondary production (He and Silliman 2016; Moore et al. 2020), community assemblage (He et al. 2015), and erosion potential (Brisson et al. 2014; Coverdale et al. 2014; Farron et al. 2020; Beheshti et al. 2021). Consumer fronts created by herbivores, specifically, can alter both the surrounding landscape and plant foundation species via their grazing (Bertness et al. 2014; He and Silliman 2016) and dwelling activities, such as burrowing (Martinetto et al. 2016; Farron et al. 2020; Xiao et al. 2020). Specifically, herbivory can alter plant functional traits (i.e., the chemical and/or morphological features of a plant), disrupting plant performance with potential feedback to ecosystem functions mediated by these traits (Lavorel 2013; Minden and Kleyer 2015; Wright et al. 2016). Yet, how herbivore fronts shape the traits and performance of foundation species in coastal vegetated ecosystems remains a distinct knowledge gap (Moore et al. 2020).

Here, we used consumer fronts created by the herbivorous purple marsh crab, *Sesarma reticulatum* (hereafter ‘*Sesarma*’) to evaluate how grazing affected the traits and performance of the smooth cordgrass, *Spartina alterniflora* (syn. *Sporobolus alterniflorus*; hereafter ‘*Spartina*’), a foundation species in US Atlantic salt marshes (Hughes et al. 2009; Vu et al. 2017; Vu and Pennings 2021). *Spartina*'s role in ecosystem functions such as sediment stabilization (Kirwan and Guntenspergen 2012), carbon accumulation (Chmura et al. 2003; Mariotti et al. 2020), and vertical accretion (FitzGerald and Hughes 2019) is mediated by its traits (e.g., stem thickness, plant height, photosynthetic capacity, number of leaves, biomass production). Thus, evaluating grazer‐driven alterations to *Spartina* traits provides insight into controls on ecosystem functioning. In addition to its direct consumption of *Spartina* above‐ and belowground biomass, *Sesarma* burrowing can resuspend consolidated sediments and stimulate decomposition by increasing soil oxygenation, both of which contribute to higher rates of erosion (Wilson et al. 2012; Vu et al. 2017; Farron et al. 2020). *Sesarma* fronts have increased in prevalence in recent decades (Crotty et al. 2020), and their top‐down control on *Spartina* biomass, together with their burrowing activities, influences geomorphology, hydrology, and vertical accretion capacity (the process by which salt marshes build elevation) (Hughes et al. 2009; Crotty et al. 2020; Williams and Johnson 2021), reducing a salt marsh's ability to keep pace with sea‐level rise (Holdredge et al. 2009; Schultz et al. 2016; Szura et al. 2017).

**FIGURE 1:**
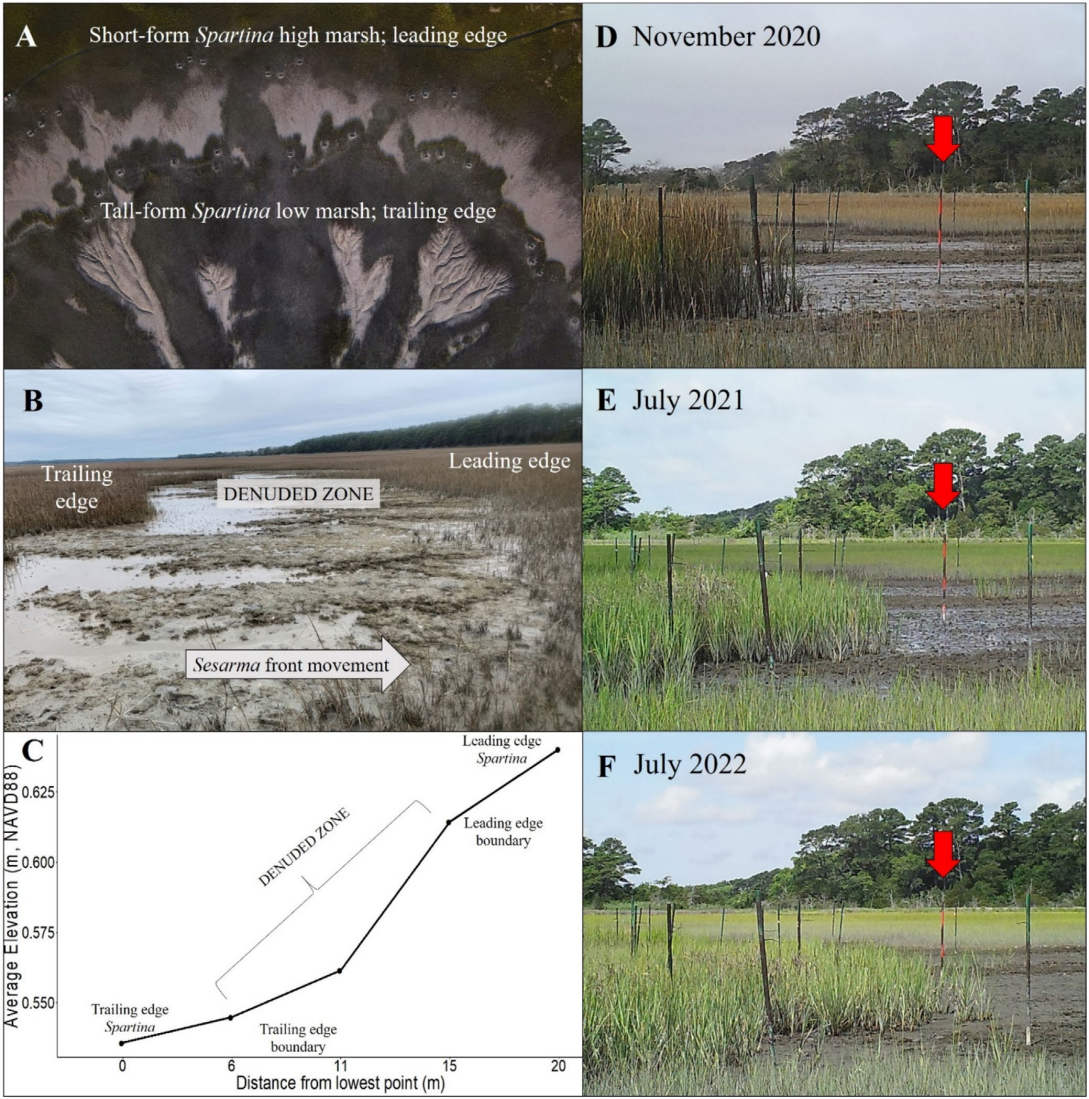
(A) Aerial photo of *Sesarma* consumer fronts on the eastern shore of Virginia with zonation labels (Photo: Aileen Devlin, Virginia Sea Grant). (B) Cross‐sectional photo of a *Sesarma* consumer front with zonation labels (Photo: Authors). (C) Elevation profile showing average elevation in meters (NAVD88) by distance from the lowest point in meters. Labels indicate distinct zonation created by the *Sesarma* consumer front. (D–F) Wildlife camera timelapse photos of consumer front movement over time.

*Sesarma* fronts form at the heads of tidal creeks (hereafter ‘creekhead’) and move directionally inland as they exhaust resources (Hughes et al. 2009; Vu and Pennings 2021; Wu et al. 2021; Figure 1A,B). The rate of front migration inland in South Carolina ranges from 1.5 to 2 m y^−1^ (Hughes et al. 2009; Wittyngham et al. 2024) and those in Georgia are migrating at approximately 1.74 m y^−1^ (Wittyngham et al. 2024). Remote sensing in a recent study found that *Sesarma* fronts in Virginia are migration at an average of 0.84 m y^−1^ and suggests that seasonal patterns in *Spartina* productivity and *Sesarma* activity may shape the rate of front migration (Wittyngham et al. 2024). Further, *Sesarma*'s combined foraging and burrowing activities lower elevation and cause the transition from high to low marsh (Vu et al. 2017; Vu and Pennings 2021; Wu et al. 2021; Wittyngham et al. 2024). Thus these fronts create three distinct zones: the leading edge of the front (i.e., ungrazed short‐form *Spartina* high marsh, hereafter ‘leading edge’), the trailing edge of the front (i.e., revegetated tall‐form *Spartina* low marsh, hereafter ‘trailing edge’), and a narrow band (10–20 m wide) of denuded mudflat in between these zones where *Sesarma* are actively burrowing and foraging (Figure 1A,B).

Using a combination of observational data and an experimental caging experiment, our objectives for this study were to: (1) quantify *Sesarma* front migration rate in the field and impacts on marsh elevation in Virginia, (2) test how direct grazing from *Sesarma* altered geomorphic processes (sediment shear strength, soil organic matter (SOM), and sediment bulk density) and *Spartina* traits within the leading edge and within the trailing edge, and (3) examine how *Sesarma*‐driven trait alterations persisted through the last 8 weeks of the growing season. We hypothesized that *Sesarma* grazing would reduce sediment shear strength, SOM, and bulk density, while negatively affecting *Spartina* traits, with trait alterations lasting through the remainder of the growing season.

## 2 Materials and Methods

### 2.1 Study System

To assess how *Sesarma* fronts influenced both the landscape and *Spartina* traits, we conducted field surveys and collections across 13 individual *Sesarma*‐impacted creekheads along the Eastern Shore of Virginia, United States (Table S1, Figure S1).

### 2.2 Marsh Elevation and Sesarma Front Movement

We used a Real Time Kinematic (RTK) Global Positioning System (GPS) to measure elevation along transects spanning from the leading edge to the trailing edge at all 13 *Sesarma*‐impacted creekheads (Table S1). Elevation was averaged from all sites to generate an elevation profile (Figure 1C).

At five creekheads (Table S1), we measured the rate of *Sesarma* front movement over time by delineating the vegetation boundaries at both the trailing and leading edges with PVC poles in July of 2020 (*n* = 15 poles per zone, per creekhead). Poles were spaced such that they encapsulated the entire length of each zone border at each creekhead. The distance from the vegetation line to the PVC poles was measured in 6‐month intervals through November of 2021. The distance from the vegetation line in November of 2020 was subtracted from the distances recorded in November of 2021 to calculate an annual rate of movement. Negative values at the leading edge indicated a retreat of vegetation (i.e., *Sesarma* front movement inland) and positive values at the trailing edge indicated revegetation. The average distance in meters of retreat and revegetation was then calculated as consumer front movement in meters per year. At the same time as pole installation, wildlife cameras (Bushnell; Overland Park, Kansas, USA) were deployed at the same five *Sesarma*‐impacted creekheads (Table S1) to visually follow consumer front movement (leading‐edge retreat, tall‐form revegetation) over time (Figure 1D–F).

### 2.3 Geomorphic Processes and Plant Traits

To experimentally test the effect of *Sesarma* grazing on geomorphic processes and *Spartina* traits, we used a block design and installed a series of exclusion (e.g., no herbivory) and inclusion (e.g., crab additions for herbivory and then removal for plant recovery) cages in the trailing‐edge and leading‐edge *Spartina* zones at eight *Sesarma*‐impacted creekheads (Table S1). The caging experiment ran for ~6 months in total, with 3 months of *Sesarma* herbivory in inclusion cages. Each creekhead had one block in the leading‐edge zone and one in the trailing‐edge zone. Each block consisted of three treatments: (1) *Sesarma* addition (hereafter ‘grazed’), (2) *Sesarma* exclusion (hereafter ‘ungrazed’), and a (3) cage control. Treatment plots were 1 m^2^, and plots within each block were 2.5 m apart. All blocks were placed 1.5 m from the edges of the *Sesarma* front to eliminate potential confounding effects. Cages were constructed of hardware cloth with 6.35 mm^2^ openings, and for grazed and ungrazed plots, caging material was dug approximately 15 cm into the sediment to prevent crab escape or entrance. Cage controls had a 15 cm tall portion removed from the bottom of the cage to allow mobile organisms to move freely. Trenches were dug around cage control plots similar to those made for the caged plots to simulate comparable levels of belowground disturbance. All cages were open at the top and a piece of aluminum flashing was attached on the inside and the outside of the uppermost 10 cm of each cage to prevent climbing organisms from entering or exiting.

At the beginning of the experiment, one open pit trap (9 cm wide × 19 cm deep) was installed in each grazed and ungrazed cage to help remove any mobile organisms (e.g., *Sesarma*, fiddler crabs). Capped pit traps were installed in cage control plots to mimic disturbance. Pit traps were emptied every other day for 2 weeks. At this point, open pit traps in grazed plots were replaced with capped pit traps and seven adult *Sesarma* (carapace width > 15 mm) were added to each grazed cage. This density is comparable to those used in a previous *Sesarma* addition study (Angelini et al. 2018), reflects the high densities of *Sesarma* seen at similar fronts in the Southeast (Hughes et al. 2009; Vu and Pennings 2021), and ensured that grazing occurred within our cages. *Sesarma* were allowed to forage for 3 months, and during this time, open pit traps in the ungrazed cages were emptied every 2 weeks. After this time, capped pit traps in the grazed plots were replaced with open pit traps to remove *Sesarma* to ensure that enough grazed plant material remained for trait analysis. Pit traps were checked daily for 1 week, and then checked every 2 weeks for the remainder of the experiment. Once *Sesarma* were removed, we counted and attached fluorescent mini zipties to the base of *Spartina* stems that had been clearly grazed by *Sesarma*.

Two weeks following *Sesarma* removal, we collected composite samples of 3–5 *Spartina* stems from each treatment plot at 2‐week intervals (began on August 2nd, 2021 and ended on September 16th, 2021; 4 time periods total) to assess trait change during the growing season. Grazed stems were collected from grazed plots and ungrazed stems were collected from all other treatment plots. All collected plants were thoroughly rinsed with DI water to remove sediments and measured for stem height and width. A penetrometer measured tissue toughness of the first six leaves (from bottom of the plant) and was averaged per stem (Failon et al. 2020). *Spartina* plants were then placed in a −80°C freezer within 3 h of collection. At the final collection of aboveground biomass (time period 4, 8‐weeks post grazing), we also destructively collected the belowground biomass of two *Spartina* stems from each plot to evaluate treatment effects.

All plants were freeze‐dried (Labconco; Kansas City, MO, USA) and ground to a fine powder using a mini Wiley Mill fitted with a 40‐mesh sieve (Thomas Scientific; Swedesboro, NJ, USA). Aboveground tissues were analyzed for carbon, nitrogen, C:N ratio, chlorophyll *a*, total phenolics, and biogenic silica. Carbon, nitrogen, and C:N ratio provide information about plant performance and nutritional content, as herbivores prefer plants with high nitrogen and low C:N ratios. Carbon and nitrogen were measured on a FlashEA elemental analyzer (Thermo Fisher Scientific; Waltham, MA, USA) and quantified using acetanilide check standards and a standard curve. C:N ratios were calculated based on these results. Chlorophyll *a* concentration, a proxy for photosynthetic capacity (Croft et al. 2017), was measured spectrophotometrically (Wellburn 1994; Warren 2008; Tran et al. 2018; Nguyen et al. 2020). To assess *Spartina*'s chemical defensive ability against herbivores, phenolic concentrations were measured using a modified Folin–Ciocalteu method (Ainsworth and Gillespie 2007; Wittyngham et al. 2019, 2023; Wittyngham 2020) and compared to a gallic acid standard curve. Biogenic silica, a structural defense against grazing, was measured using a wet chemical alkaline extraction (DeMaster 1981; Conley and Schelske 2002) and then transferred to the Virginia Institute of Marine Science (VIMS) Analytical Laboratory for measurement of dissolved silica concentrations (Strickland and Parsons 1972).

At the end of the experiment, a handheld shear vane (AMS Inc.; American Falls, ID, USA) fitted with a 25.4 × 50.8 mm vane was used to test sediment shear strength in all plots (grazed, ungrazed, cage control; *n* = 8 per treatment, per zone). To measure soil organic matter (SOM) and bulk density, we collected one 30‐cm deep core via a Russian peat borer (Forestry Suppliers Inc.; Jackson, MS, USA) in all plots (grazed, ungrazed, cage control; *n* = 8 per treatment, per zone). SOM was calculated using standard loss on ignition techniques, and bulk density was calculated as the mass of the dry sample divided by the borer volume (Wilson et al. 2012).

### 2.4 Statistics

All statistical analyses were conducted in RStudio version 4.2.2 (R Core Team 2022).

Two‐way ANOVAs with main effects of treatment (i.e., grazed, ungrazed, cage control) and spatial location (i.e., creekhead) were used to quantify differences in sediment shear strength, SOM, bulk density, above‐ and belowground biomass, and root: shoot ratios. For each of these responses, separate ANOVAs were conducted for trailing edge and leading edge variables (Table S2). To assess the effects of *Sesarma* grazing on plant traits, a repeated measures MANOVA was conducted with spatial location (i.e., creekhead) and treatment (i.e., grazed, ungrazed, cage control) as main factors, and sampling period as the repeated measures factor. Separate MANOVAs were conducted for trailing edge and leading edge variables. All plant traits were combined into a single response variable (cbind function, base R) prior to running the MANOVAs. A MANOVA was used because multiple traits were measured on a single composite sample (*n* = 3–5 stems); thus, responses were assessed in a single model to avoid inflating our Type I error. The blocking factor “spatial location” (i.e., creekhead) was included in all statistical models to reduce unexplained variation. All response variables were tested to meet model assumptions, and only carbon, nitrogen, and chlorophyll *a* were log transformed to meet the assumption of normality. Interaction terms were included in all models, and complete statistical reporting is available in Table S2.

## 3 Results

### 3.1 Marsh Elevation and Sesarma Front Movement

There was an average drop in elevation of 10.5 ± 0.5 cm (mean ± standard error) from the leading edge to the trailing edge, with the steepest scarp occurring within the denuded band of mudflat separating the two zones (Figure 1C). The *Sesarma* fronts at the five evaluated creekheads moved inland at an average rate of 0.88 ± 0.12 m y^−1^ (Figure 1D–F), similar to findings from remote sensing work, which calculated an average migration rate of 0.84 m y^−1^ in the same region (Wittyngham et al. 2024). At these sites, the leading edge retreated at an average rate of 1.07 m ± 0.18 m y^−1^, whereas the trailing edge revegetated at an average rate of 0.69 ± 0.05 m y^−1^.

### 3.2 Geomorphic Processes & Plant Traits

**FIGURE 2:**
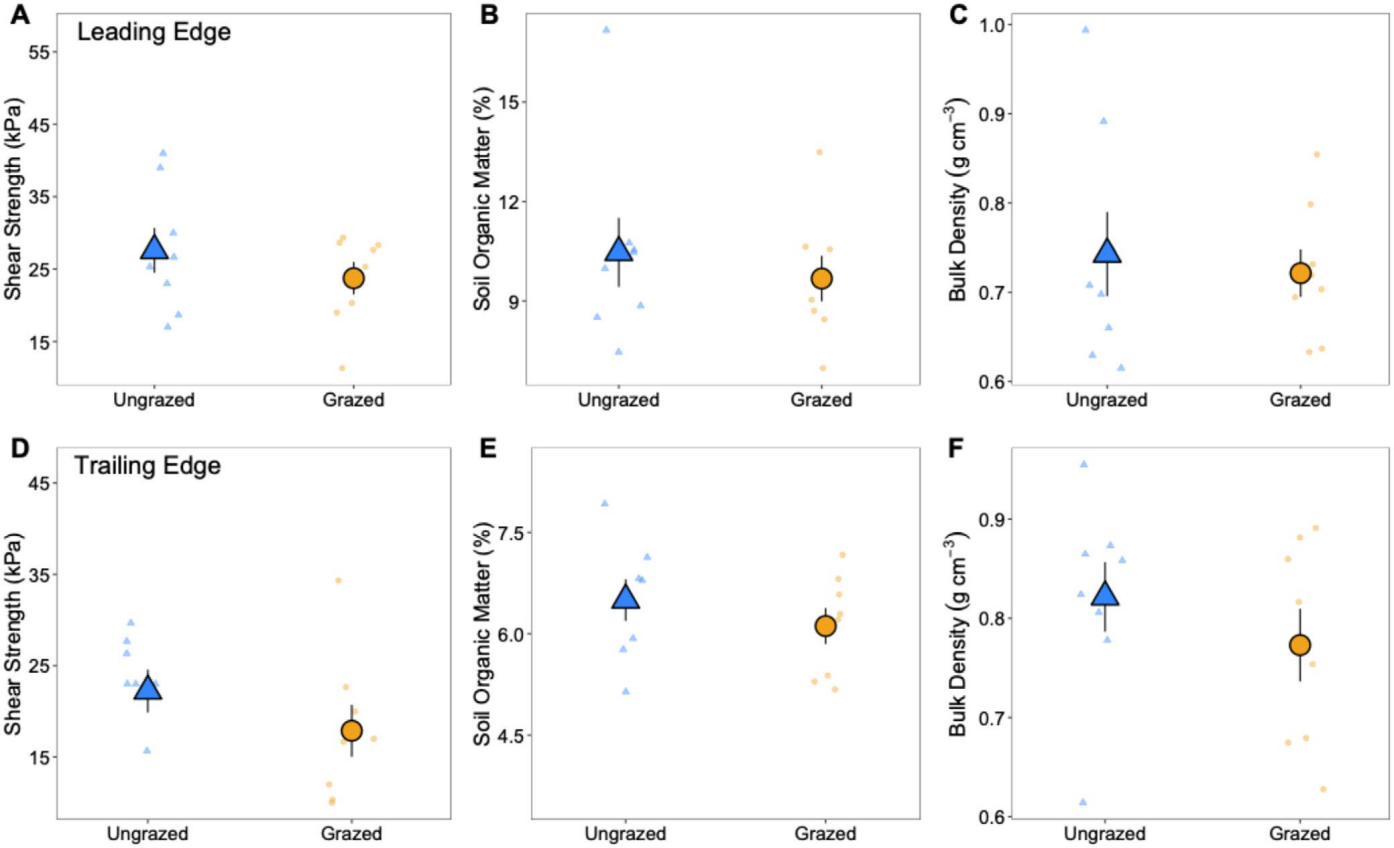
Average shear strength*, soil organic matter (SOM), and sediment bulk density of ungrazed plots (blue triangles) and grazed plots (yellow circles) at the short‐form *Spartina* leading edge (panels A, B, and C) and at the tall‐form *Spartina* trailing edge (panels D, E, and F). Large symbols represent mean ± 1 standard error overlaid on raw data. An asterisk (*) next to a response variable in this caption indicates a significant difference (*p* < 0.05) between ungrazed and grazed treatments.

There were no effects of caging on geomorphic processes or *Spartina* trait responses (i.e., no significant differences between treatment plots and cage controls), thus the results presented are for grazed and ungrazed plots only. All percent difference calculations are based on averaged trait values across all sampling time periods. At the trailing edge, grazing caused a 29% decline in sediment shear strength in comparison to ungrazed plots (ANOVA, F_3,43_ = 2.015, *p* = 0.0409), and grazing had no effect on sediment shear strength at the leading edge (ANOVA, F_3,44_ = 0.7891, *p* = 0.1826) (Figure 2A,D). Grazing had no effect on soil organic matter (SOM) (leading edge: ANOVA, F_15,48_ = 0.7202, *p* = 0.5861; trailing edge: ANOVA, F_15,48_ = 1.006, *p* = 0.3449) or bulk density (leading edge: ANOVA, F_15,48_ = 0.3310, *p* = 0.8160; trailing edge: ANOVA, F_15,48_ = 0.3916, *p* = 0.5501), regardless of zone (Figure 2B,C,E,F).

Ungrazed cages were nearly 100% effective at excluding *Sesarma* at both the leading and trailing edge, with 4 total *Sesarma* crabs removed from 3 (two leading edge, one trailing edge) of the 16 ungrazed cages at the first pit trap check. The remaining 13 cages had no *Sesarma* present. For the following 6 pit trap checks, there were 0 adult *Sesarma* found in any of the 16 ungrazed cages. There were clear signs of *Sesarma* herbivory in grazed cages, and grazing intensity (i.e., number of grazed stems) increased over time. Immediately following *Sesarma* addition, there were an average of eight grazed stems per square meter. In the following bi‐monthly counts, there were an average of 13, 12, and 23 grazed stems per square meter. In contrast, there was an average of 1 grazed stem per square meter in the ungrazed cages, and this number did not increase over time. Further, grazed cages had an average of 6 *Sesarma* burrows per square meter, whereas ungrazed cages had an average of < 1 burrow per square meter.

**FIGURE 3:**
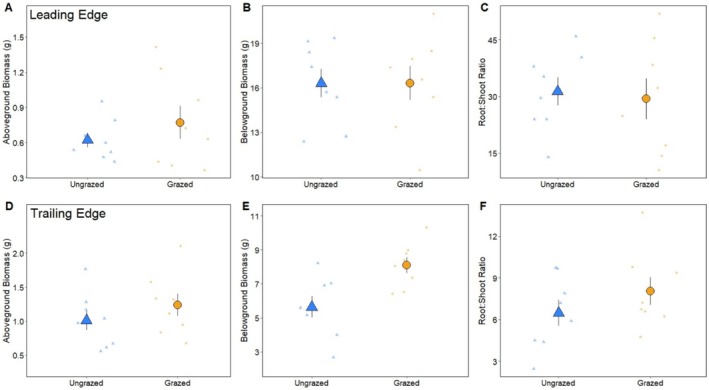
Average aboveground biomass, belowground biomass, and root: Shoot ratios of ungrazed *Spartina* (yellow triangles) and grazed *Spartina* (blue circles) at the short‐form *Spartina* leading edge (panels A–C) and at the tall‐form *Spartina* trailing edge (panels D, E, and F). Large symbols represent mean ± 1 standard error overlaid on raw data. An asterisk (*) next to a panel label in this caption indicates a significant difference (*p* < 0.05) between ungrazed and grazed treatments.

At the leading edge, there was a significant interaction of treatment and spatial location (i.e., creekhead) on aboveground biomass (ANOVA, F_3,28_ = 3.7610, *p* = 0.0116), and no effect of any factor on belowground biomass (ANOVA, F_3,28_ = 0.0852, *p* = 0.9968) or root: shoot ratio (ANOVA, F_3,28_ = 1.3190, *p* = 0.7308) (Figure 3A–C). At the trailing edge, although aboveground biomass (ANOVA, F_3,27_ = 0.9850, *p* = 0.2795) and root: shoot ratio were unaffected by treatment (ANOVA, F_3,27_ = 2.523, *p* = 0.1856), *Sesarma* grazing caused a 39% increase in *Spartina* belowground biomass (ANOVA, F_3,28_ = 4.1680, *p* = 0.0032) (Figure 3D–F).

**FIGURE 4:**
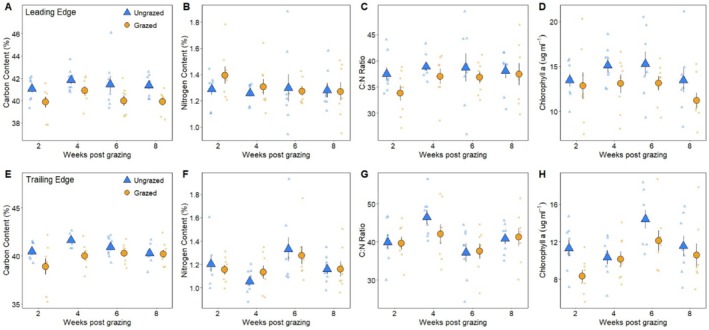
Average carbon content, nitrogen content, C:N ratio, and chlorophyll *a* concentrations of ungrazed *Spartina* (yellow triangles) and grazed *Spartina* (blue circles) over time at the short‐form *Spartina* leading edge (panels A*, B, C, and D*) and at the tall‐form *Spartina* trailing edge (panels E, F, G, and H). Large symbols represent mean ± 1 standard error overlaid on raw data. An asterisk (*) next to a panel label in this caption indicates a significant difference (*p* < 0.05) between ungrazed and grazed treatments.

**FIGURE 5:**
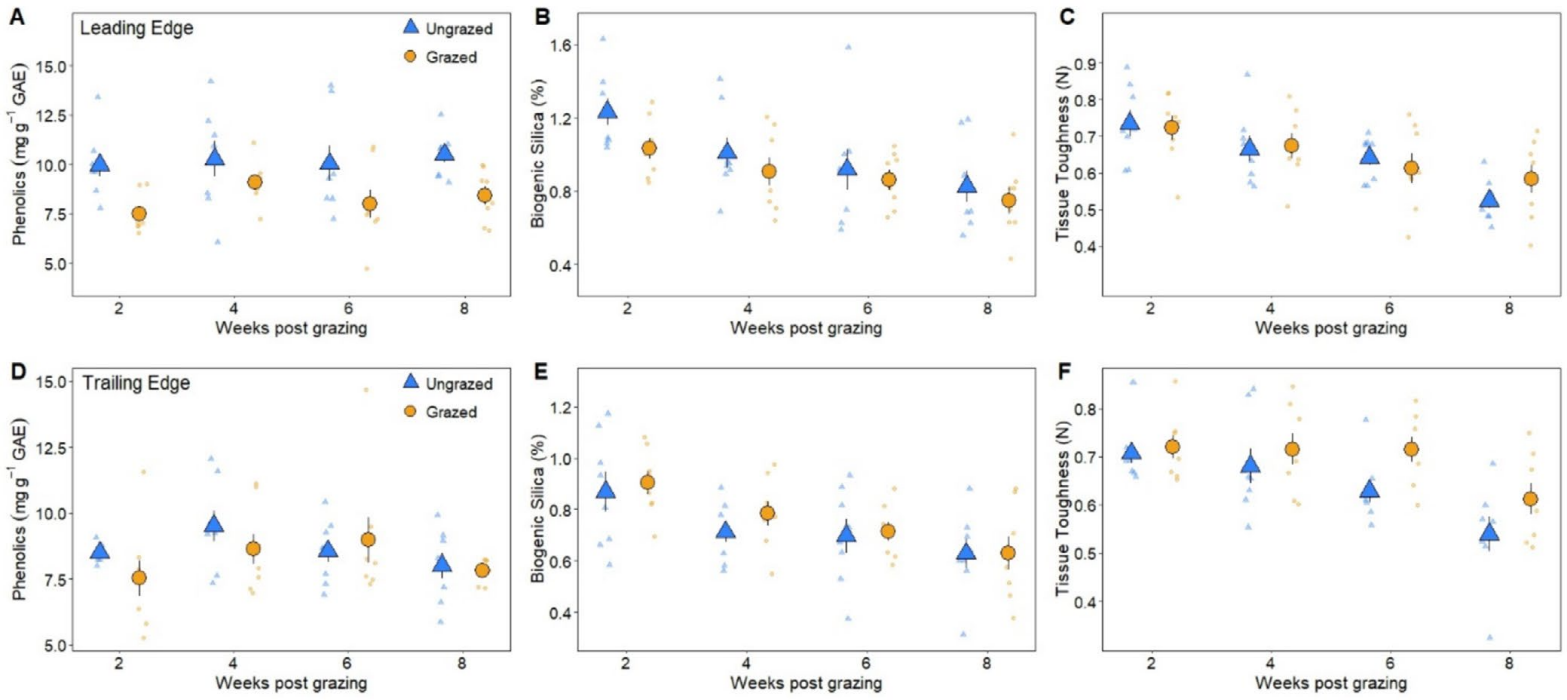
Average phenolic concentrations, biogenic silica, and tissue toughness of ungrazed *Spartina* (yellow triangles) and grazed *Spartina* (blue circles) over time at the short‐form *Spartina* leading edge (panels A*, B*, and C) and at the tall‐form *Spartina* trailing edge (panels D, E, and F). Large symbols represent mean ± 1 standard error overlaid on raw data. An asterisk (*) next to a panel label in this caption indicates a significant difference (*p* < 0.05) between ungrazed and grazed treatments.

*Sesarma* grazing had a significant negative effect on *Spartina* traits at the leading edge. For growth traits, grazed *Spartina* had 3% less carbon (RM (repeated measures) MANOVA, F_1_ = 14.8367, *p* = 0.0005), and 13% less chlorophyll *a* (RM MANOVA, F_1_ = 7.6544, *p* = 0.0093) when compared to ungrazed plants (Figure 4A,D). Chlorophyll *a* concentrations varied by creekhead (RM MANOVA, F_7_ = 4.2643, *p* = 0.0020), although there was not a significant interaction between treatment and creekhead (RM MANOVA, F_7_ = 1.4686 *p* = 0.2136). There was a significant interaction of treatment and creekhead on nitrogen content (RM MANOVA, F_7_ = 2.8018, *p* = 0.0216) and C:N ratio (RM MANOVA, F_7_ = 2.5272, *p* = 0.0345), thus main effects were not interpreted further for these responses (Figure 4B,C). For defensive traits, *Sesarma* grazing at the leading edge caused a 21% decline in phenolic concentrations (RM MANOVA, F_1_ = 20.4079, *p* < 0.0001) and a 12% reduction in biogenic silica (RM MANOVA, F_1_ = 10.1433, *p* = 0.0032) (Figure 5A,B). Biogenic silica also varied by sampling period (RM MANOVA, F_1_ = 48.6449, *p* < 0.0001), with declines in concentration over time, and creekhead (RM MANOVA, F_7_ = 13.9683, *p* < 0.0001), although there were no significant interactions between factors. There was a significant interaction between treatment, sampling period, and creekhead on *Spartina* tissue toughness (RM MANOVA, F_1_ = 2.2392, *p* = 0.0568) (Figure 5C), thus main effects were not interpreted for this response.

In contrast, *Sesarma* grazing had few impacts on *Spartina* at the trailing edge. There was a significant interaction of treatment and sampling period on carbon content (RM MANOVA, F_1_ = 4.0884, *p* = 0.0516), with grazing decreasing carbon content initially (2‐weeks post grazing), and no differences between grazed and ungrazed plants at 4, 6, and 8 weeks post grazing, suggesting rapid recovery (Figure 4E). Grazing had no effect on chlorophyll *a* concentrations (RM MANOVA, F_1_ = 3.7053, *p* = 0.0632), nitrogen content (RM MANOVA, F_1_ = 00012, *p* = 0.9731) or C:N ratio (RM MANOVA, F_1_ = 0.2772, *p* = 0.6022) at the trailing edge (Figure 4F–H). For defensive traits, only creekhead had a significant effect on phenolics (RM MANOVA, F_7_ = 4.4309, *p* = 0.0015) (Figure 5D), and there were significant interactions between treatment, sampling period, and creekhead for biogenic silica (RM MANOVA, F_7_ = 2.2205, *p* = 0.0587) and between treatment and creekhead for *Spartina* tissue toughness (RM MANOVA, F_7_ = 3.0453, *p* = 0.0142); thus, main effects were not interpreted further for these responses (Figure 5E,F).

## 4 Discussion

Consumer fronts can shape primary production, community composition, and ecosystem stability as high densities of consumers move through the landscape exhausting resources (Silliman et al. 2013). Consumer fronts created by herbivores, in particular, not only shape the landscape, but can also alter the traits of plant foundation species, which are inherently linked to ecosystem functioning. Yet, herbivore‐driven plant trait alterations remain understudied. We addressed this knowledge gap by examining how *Sesarma* fronts are affecting saltmarsh ecosystems at both the landscape scale (e.g., elevation change, front migration rate) and at the individual plant scale in the US mid‐Atlantic region. Our findings show that *Sesarma* fronts lower elevation as they migrate inland (Figure 1C), allowing for the revegetation of tall‐form *Spartina* at the trailing edge, a finding similar to previous work on *Sesarma* fronts (Vu et al. 2017; Vu and Pennings 2021; Wu et al. 2021; Wittyngham et al. 2024). However, the rate of vegetation retreat at the leading edge is greater than the rate of revegetation, potentially enhancing creek elongation and expansion and intensifying increases in creek growth already caused by sea level‐driven changes in tidal range. *Sesarma* grazing decreased sediment shear strength at the trailing edge, although it had no effect on SOM or bulk density in either zone. *Sesarma* grazing had differential impacts on *Spartina* traits, with plants at the leading edge having reduced growth (e.g., carbon, chlorophyll *a*) and defensive traits (e.g., phenolics, biogenic silica) in response to grazing, and these trait changes persisted for 8 weeks. Interestingly, plants at the trailing edge were resistant to herbivore disturbance, and grazing increased plant belowground biomass production, which could promote ecosystem stability.

### 4.1 Marsh Elevation and Sesarma Front Movement

On average, *Sesarma* fronts caused a 10.5 cm drop in elevation from the leading‐edge boundary to the trailing‐edge boundary (Figure 1C), which is more than three times greater than the average change in elevation between high and low marsh zones at ungrazed creekheads along the Eastern Shore of Virginia (3.1 cm; Messerschmidt, T.C., *unpublished data*). We found that *Sesarma* fronts in Virginia are moving at an average rate of ~0.9 m yr.^−1^, which is two times slower than others' findings in Georgia (Vu and Pennings 2021) and South Carolina (Hughes et al. 2009). This could ultimately be a function of seasonality, with Virginia marshes having distinct seasons for *Spartina* growth and *Sesarma* grazing, limiting the time for consumer front development. Further, at our study sites, the rate of short‐form *Spartina* retreat at the leading edge is 43% faster on average than the rate of tall‐form *Spartina* revegetation at the trailing edge, suggesting that if conditions remain steady over time, the width of the front may widen, with potential feedback to geomorphic and hydrological conditions. To our knowledge, there have been no documented occurrences of these *Sesarma*‐driven impacts reverting. Across geographic regions where *Sesarma* fronts have been studied, once elevation has been lowered and the low marsh established with revegetated tall‐form *Spartina*, there is no return to high marsh conditions (Hughes et al. 2009; Vu and Pennings 2021; Wu et al. 2021; Wittyngham et al. 2024).

### 4.2 Geomorphic Processes and Plant Traits

We found that *Sesarma* grazing only decreased sediment shear strength at the trailing edge (Figure 2D), similar to a previous study (Wilson et al. 2012). However, grazing had no effect on sediment shear strength at the leading edge (Figure 2A) and did not influence SOM or sediment bulk density in either zone (Figure 2B,C,E,F). *Sesarma* have been shown to negatively impact SOM and bulk density in other regions via increased decomposition (Wilson et al. 2012) and sediment excavation (Vu et al. 2017), respectively. One possible explanation for our lack of response could be that *Sesarma* were removed from grazed treatment plots after 3 months, which may not have been enough time for these longer‐term processes to be affected.

*Sesarma* fronts negatively affected *Spartina* traits at the leading edge but had little to no impact at the trailing edge, and even a positive effect on tall‐form belowground biomass production (Figure 3E). Through their direct grazing, *Sesarma* reduced *Spartina* performance at the leading edge via alterations in its growth (e.g., lowered carbon, chlorophyll *a*; Figure 4A,D) and defensive traits (e.g., decreased phenolics, biogenic silica; Figure 5A,B) when compared to ungrazed stems. This pattern opposes what plant defense theory predicts, as we would expect grazing to enhance plant defenses, such as when gypsy‐moth herbivory increased the toughness and tannin content of oak tree leaves (Lance et al. 1986) and limited subsequent grazing. These plant trait changes persisted throughout the final 8 weeks of the growing season, suggesting limited or slow recovery. These trait alterations have important implications for front propagation, as reduced performance and weakened defensive ability at the leading edge may increase *Spartina* susceptibility to grazing (from *Sesarma* and/or other invertebrate herbivores), contributing to continued front migration inland.

The only measured *Spartina* trait at the trailing edge that was significantly influenced by grazing was carbon content (Figure 4E), although this varied by sampling period. At 2 weeks post herbivory, the carbon content of grazed plants was significantly lower than that of ungrazed plants; however, by 4 weeks post grazing, there were no significant differences between grazed and ungrazed plants. This rapid recovery of carbon content in tall‐form *Spartina* at the trailing edge was not seen in short‐form *Spartina* at the leading edge (Figure 4A). The resistance and quick recovery of trailing edge tall‐form *Spartina* to herbivore perturbation is most likely an indirect effect of the enhanced environmental conditions and increased resources associated with elevated tidal flushing typical of low marsh zones (Friedrichs and Perry 2001; Morris et al. 2002). Interestingly, the lowered elevation and subsequent changes in hydrology and sediment properties are resultant from *Sesarma* front propagation (Hughes et al. 2009; Wilson et al. 2012; Crotty et al. 2020). Combined with grazing‐induced increased belowground biomass production at the trailing edge, *Sesarma* fronts are shaping marsh stability and resilience to sea‐level rise.

*Sesarma*'s consumption of *Spartina*, together with its burrowing, influence a salt marsh's geomorphology, hydrology, erodibility, and vertical accretion capacity (Hughes et al. 2009; Wilson et al. 2012; Vu et al. 2017; Farron et al. 2020; Crotty et al. 2020; Williams and Johnson 2021), potentially reducing its ability to keep pace with sea‐level rise (Holdredge et al. 2009; Schultz et al. 2016; Szura et al. 2017). We built upon this previous work and found that *Sesarma* grazing indirectly (e.g., modified elevation) and directly (e.g., grazing) altered the traits and performance of *Spartina*, a foundation species critical for saltmarsh persistence. Combined, our results suggest that *Sesarma* grazing results in poor plant performance and defensive ability at the leading edge, potentially promoting front migration inland, and resistant *Spartina* with enhanced belowground biomass production at the trailing edge, aiding in marsh resilience to sea‐level rise following intense grazing disturbance.

In some instances, unconstrained consumer fronts can influence ecosystem resilience and cause permanent state change (Silliman et al. 2013; Vu and Pennings 2021), such as the shift from healthy kelp forests into urchin barrens caused by overgrazing (Ling et al. 2009). Further, many consumers such as insects (Lejeune et al. 2005; Birt and Coulson 2015), invertebrates (Kroon et al. 2021), and microbes (Muller and van Woesik 2012) form consumer fronts worldwide, and these consumers are often foraging on plant foundation species, similar to our work presented here. Food quality can be a key determinant of mobile consumer distribution in other ecosystems, such as geese in the *Carex* spp. meadows of Eastern Asia (Zhang et al. 2020), suggesting that altered plant traits and performance caused by grazing may be a common occurrence in other ecosystems. Thus, it is critical to evaluate both landscape and plant trait change in the context of consumer fronts to better predict ecosystem response and recovery.

## Author Contributions

**Serina S. Wittyngham:** conceptualization (equal), data curation (lead), formal analysis (lead), investigation (lead), methodology (equal), visualization (lead), writing – original draft (lead), writing – review and editing (lead). **David S. Johnson:** conceptualization (equal), funding acquisition (lead), investigation (supporting), methodology (equal), resources (lead), supervision (lead), writing – review and editing (supporting).

## Conflicts of Interest

The authors declare no conflicts of interest.

## Supporting information


**Figure S1.** (A) Map of creekheads used in this study, with scale bar in meters in the bottom left of the map. (B) Inset map of larger study region with a pin marking the Eastern Shore of Virginia, USA where this study occurred.

**Table S1.** Longitude, latitude, and variables measured for each of the 13 creekheads used in our study.

**Table S2.** Complete statistical report for all models and responses. An asterisk (*) indicates significance at an alpha of 0.05.

## Data Availability

All associated data and scripts have been archived with the Virginia Coast Reserve (VCR) Long‐Term Ecological Research (LTER) and made publicly available through the Environmental Data Initiative (EDI) at: https://doi.org/10.6073/pasta/8afcae9394cee77fb6f6ee3e739fd38d.
