# Supplementary material for: Herbivore Fronts Shape Saltmarsh Plant Traits and Performance
Source: Ecol Evol. 2025 Apr 25;15(4):e71360. doi: 10.1002/ece3.71360 (PMC12022777; doi:10.1002/ece3.71360)

**Manuscript title:** Herbivore fronts shape saltmarsh plant traits and performance

**Journal:** *Ecology & Evolution*

**Figure S1.** (A) Map of creekheads used in this study, with scale bar in meters in the bottom left of the map. (B) Inset map of larger study region with a pin marking the Eastern Shore of Virginia, U.S. where this study occurred.


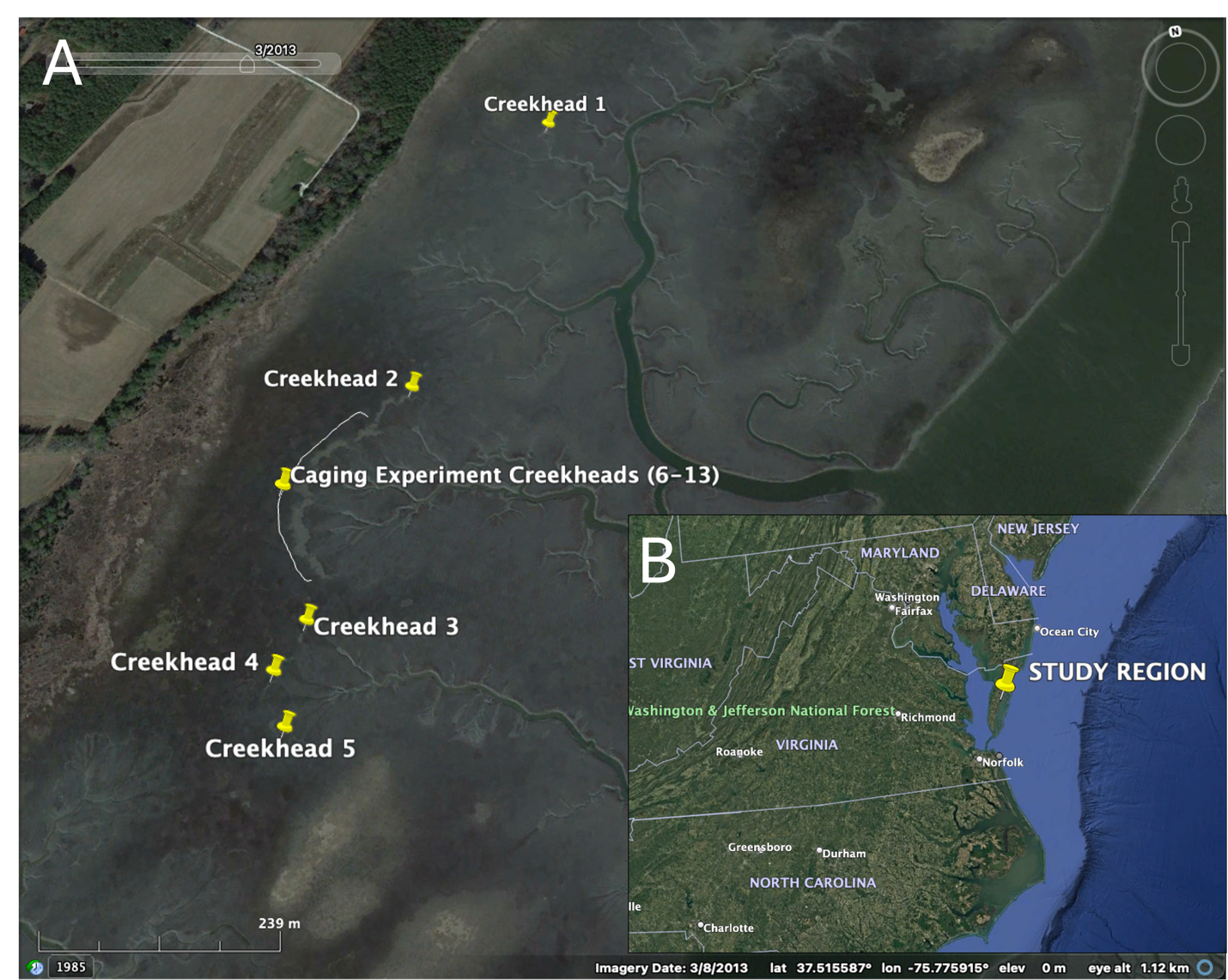

Supplement: Supplementary file 1 — Figure S1. (A) Map of creekheads used in this study, with scale bar in meters in the bottom left of the map. (B) Inset map of larger study region with a pin marking the Eastern Shore of Virginia, USA where this study occurred. [file ECE3-15-e71360-s001.docx]
