# Supplementary material for: Herbivore Fronts Shape Saltmarsh Plant Traits and Performance
Source: Ecol Evol. 2025 Apr 25;15(4):e71360. doi: 10.1002/ece3.71360 (PMC12022777; doi:10.1002/ece3.71360)
Supplement: Supplementary file 2 — Table S1. Longitude, latitude, and variables measured for each of the 13 creekheads used in our study. [file ECE3-15-e71360-s002.docx]

**Manuscript title:** Herbivore fronts shape saltmarsh plant traits and performance

**Journal:** *Ecology and Evolution*

**Table S1.** Longitude, latitude, and variables measured for each of the thirteen creekheads used in our study.

| **Creekhead Number** | **Latitude** | **Longitude** | **Variables Measured** |
| --- | --- | --- | --- |
| 1 | 37.520921°N | -75.781983°W | Rate of movement, wildlife camera, elevation |
| 2 | 37.517749°N | -75.784335°W | Rate of movement, wildlife camera, elevation |
| 3 | 37.515381°N | -75.785890°W | Rate of movement, wildlife camera, elevation |
| 4 | 37.514927°N | -75.786319°W | Rate of movement, wildlife camera, elevation |
| 5 | 37.514373°N | -75.786216°W | Rate of movement, wildlife camera, elevation |
| 6 | 37.517395°N | -75.784761°W | Caging experiment, elevation, SOM, bulk density, shear strength |
| 7 | 37.517387°N | -75.785033°W | Caging experiment, elevation, SOM, bulk density, shear strength |
| 8 | 37.517330°N | -75.785241°W | Caging experiment, elevation, SOM, bulk density, shear strength |
| 9 | 37.517289°N | -75.785431°W | Caging experiment, elevation, SOM, bulk density, shear strength |
| 10 | 37.516855°N | -75.785772°W | Caging experiment, elevation, SOM, bulk density, shear strength |
| 11 | 37.516712°N | -75.785809°W | Caging experiment, elevation, SOM, bulk density, shear strength |
| 12 | 37.516381°N | -75.785839°W | Caging experiment, elevation, SOM, bulk density, shear strength |
| 13 | 37.516013°N | -75.785662°W | Caging experiment, elevation, SOM, bulk density, shear strength |
