# Supplementary material for: Herbivore Fronts Shape Saltmarsh Plant Traits and Performance
Source: Ecol Evol. 2025 Apr 25;15(4):e71360. doi: 10.1002/ece3.71360 (PMC12022777; doi:10.1002/ece3.71360)
Supplement: Supplementary file 3 — Table S2. Complete statistical report for all models and responses. An asterisk (*) indicates significance at an alpha of 0.05. [file ECE3-15-e71360-s003.docx]

**Manuscript title:** Herbivore fronts shape saltmarsh plant traits and performance

**Journal:** *Ecology and Evolution*

**Table S2.** Complete statistical report for all models and responses. An asterisk (*) indicates significance at an alpha of 0.05.

| **ANOVA RESULTS** | **Factors & p-values** | | |  |
| --- | --- | --- | --- | --- |
| **Response (Leading Edge)** | **Treatment** | **Creekhead** | **Treatment x Creekhead** | **F statistic and degrees of freedom (DF)** |
| Aboveground biomass | 0.2056 | 0.1401 | 0.0116* | 3.7610 on 3 and 28 DF |
| Belowground biomass | 0.9968 | 0.6620 | 0.8079 | 0.0852 on 3 and 28 DF |
| Root:Shoot ratio | 0.7308 | 0.3176 | 0.1053 | 1.3190 on 3 and 28 DF |
| Soil organic matter (SOM) | 0.5861 | 0.2973 | 0.9681 | 0.7202 on 15 and 48 DF |
| Bulk density | 0.8160 | 0.8701 | 0.9668 | 0.3310 on 15 and 48 DF |
| Sediment shear strength | 0.1826 | 0.5747 | 0.6460 | 0.7891 on 3 and 44 DF |
| **Response (Trailing Edge)** | **Treatment** | **Creekhead** | **Treatment x Creekhead** | **F statistic and degrees of freedom (DF)** |
| Aboveground biomass | 0.2795 | 0.9140 | 0.2001 | 0.9850 on 3 and 27 DF |
| Belowground biomass | 0.0032* | 0.4390 | 0.2375 | 4.1680 on 3 and 28 DF |
| Root:Shoot ratio | 0.1856 | 0.1741 | 0.0625 | 2.5230 on 3 and 27 DF |
| Soil organic matter (SOM) | 0.3449 | 0.1870 | 0.8177 | 1.0060 on 15 and 48 DF |
| Bulk density | 0.5501 | 0.8067 | 0.9676 | 0.3916 on 15 and 48 DF |
| Sediment shear strength | 0.0409* | 0.4545 | 0.1641 | 2.0150 on 3 and 43 DF |

| **MANOVA RESULTS** | **Factors,P-values, F-statistic, and Degrees of Freedom** | | | | | | |
| --- | --- | --- | --- | --- | --- | --- | --- |
| **PLANT TRAITS (Leading Edge)** | **Treatment**  **(p-value,**  **F value_DF_)** | **Sampling Period** | **Creekhead (Block)** | **Treatment X Sampling Period** | **Sampling Period X Creekhead** | **Treatment X Creekhead** | **Treatment x Sampling Period x Creekhead** |
| Carbon | 0.0005*, 14.8367_1_ | 0.9532, 0.0035_1_ | 0.1184, 1.8155_7_ | 0.7016, 0.1494_1_ | 0.8744, 0.4325_7_ | 0.7257, 0.6323_7_ | 0.6485, 0.7293_7_ |
| Nitrogen | 0.4246, 0.6542_1_ | 0.2414, 1.4247_1_ | 0.1089, 1.8640_7_ | 0.2454, 1.4004_1_ | 0.7640, 0.5836_7_ | 0.0216*, 2.8018_7_ | 0.8058, 0.5292_7_ |
| C:N | 0.0781, 3.3126_1_ | 0.2417, 1.4228_1_ | 0.3749, 1.1203_7_ | 0.3549, 0.8812_1_ | 0.9176, 0.3618_7_ | 0.0345*, 2.5272_7_ | 0.8850, 0.4162_7_ |
| Chlorophyll a | 0.0093*, 7.6544_1_ | 0.4291, 0.6414_1_ | 0.0020*, 4.2643_7_ | 0.6009, 0.2791_1_ | 0.6202, 0.7653_7_ | 0.2136, 1.4686_7_ | 0.6826, 0.6864_7_ |
| Phenolics | < 0.0001*, 20.4079_1_ | 0.4543, 0.5739_1_ | 0.3840, 1.1047_7_ | 0.9404, 0.0057_1_ | 0.2211, 1.4482_7_ | 0.2198, 1.4515_7_ | 0.2211, 1.4482_7_ |
| Tissue toughness | 0.7539, 0.1000_1_ | < 0.0001*, 49.3877_1_ | 0.0004*, 5.4347_7_ | 0.2567, 1.3337_1_ | 0.6744, 0.6967_7_ | 0.2818, 1.3005_7_ | 0.0568*, 2.2392_7_ |
| Biogenic silica | 0.0032*, 10.1433_1_ | < 0.0001*, 48.6449_1_ | < 0.0001*, 13.9683_7_ | 0.1903, 1.7905_1_ | 0.4096, 1.0623_7_ | 0.2381, 1.4035_7_ | 0.5810, 0.81607 |
| **PLANT TRAITS (Trailing Edge)** | **Treatment** | **Sampling Period** | **Creekhead (Block)** | **Treatment X Sampling Period** | **Sampling Period X Creekhead** | **Treatment X Creekhead** | **Treatment x Sampling Period x Creekhead** |
| Carbon | 0.0022*, 11.0405_1_ | 0.3180, 1.0290_1_ | 0.5995, 0.7918_7_ | 0.0516*, 4.0884_1_ | 0.2708, 1.3251_7_ | 0.0913, 1.9654_7_ | 0.0670, 2.1439_7_ |
| Nitrogen | 0.9731, 0.0012_1_ | 0.5940, 0.2899_1_ | 0.6992, 0.6656_7_ | 0.9959, 0.0000_1_ | 0.9693, 0.2476_7_ | 0.9943, 0.1393_7_ | 0.8888,  0.4102_7_ |
| C:N | 0.6022, 0.2772_1_ | 0.7336, 0.1178_1_ | 0.6229, 0.7617_7_ | 0.6876, 0.1646_1_ | 0.9387, 0.3214_7_ | 0.9435, 0.3114_7_ | 0.9768, 0.2238_7_ |
| Chlorophyll a | 0.0632, 3.7053_1_ | 0.0867, 3.1238_1_ | 0.4148, 1.0539_7_ | 0.5794, 0.3135_1_ | 0.9956, 0.1276_7_ | 0.9304, 0.3379_7_ | 0.8711, 0.4375_7_ |
| Phenolics | 0.2354, 1.4625_1_ | 0.6115, 0.2631_1_ | 0.0015*, 4.4309_7_ | 0.2789, 1.2130_1_ | 0.9067, 0.3808_7_ | 0.2247, 1.4385_7_ | 0.4708, 0.9679_7_ |
| Tissue toughness | 0.0020*, 11.2833_1_ | < 0.0001*, 41.7688_1_ | 0.0002*, 5.9300_7_ | 0.0917, 3.0239_1_ | 0.1092, 1.8625_7_ | 0.0143*, 3.0453_7_ | 0.9111, 0.3732_7_ |
| Biogenic silica | 0.3071, 1.0773_1_ | < 0.0001*, 35.4314_1_ | 0.0012*, 4.5915_7_ | 0.5833, 0.3071_1_ | 0.1662, 1.6175_7_ | 0.1323, 1.7509_7_ | 0.0587*, 2.2205_7_ |
